# Xanthenedione Derivatives, New Promising Antioxidant and Acetylcholinesterase Inhibitor Agents

**DOI:** 10.3390/molecules19068317

**Published:** 2014-06-19

**Authors:** Ana M. L. Seca, Stephanie B. Leal, Diana C. G. A. Pinto, Maria Carmo Barreto, Artur M. S. Silva

**Affiliations:** 1 DCTD, University of Azores, Rua Mãe de Deus, Ponta Delgada 9501-801, Portugal; 2 Department of Chemistry & QOPNA, University of Aveiro, Campus de Santiago, Aveiro 3810-193, Portugal; 3 CIRN, University of Azores, Ponta Delgada 9501-801, Portugal

**Keywords:** xanthene-1,9(2*H*)-diones, scavenging activity, reduction power, acetylcholinesterase inhibitors, xanthones, 3-cinnamoyl-5-hydroxy-2-styrylchromones

## Abstract

Natural and synthetic xanthone derivatives are well-known for their ability to act as antioxidants and/or enzyme inhibitors. This paper aims to present a successful synthetic methodology towards xanthenedione derivatives and the study of their aromatization to xanthones. Additionally their ability to reduce Fe(III), to scavenge DPPH radicals and to inhibit AChE was evaluated. The results demonstrated that xanthenedione derivative **5e**, bearing a catechol unit, showed higher reduction capacity than BHT and similar to quercetin, strong DPPH scavenging activity (EC_50_ = 3.79 ± 0.06 µM) and it was also showed to be a potent AChEI (IC_50_ = 31.0 ± 0.09 µM) when compared to galantamine (IC_50_ = 211.8 ± 9.5 µM).

## 1. Introduction

Antioxidants are necessary to control degenerative reactions produced by reactive oxygen and nitrogen species. These species are involved in several ailments including cancer, heart diseases and apparently Alzheimer’s disease (AD) [1]. Alzheimer’s disease remains a mystery and a source of several discussions [2], but there is evidence that it is related with oxidative stress [3], as well as with cholinesterase activity [4]. Although the current therapy used for AD is based on acetylcholinesterase (AChE) inhibitors [5,6] it seems that other possibilities cannot be rejected. Thus antioxidants that are considered as a prime target for potential chemoprevention of several pathologies may also become important phytochemicals against degenerative diseases such as AD.

**Figure 1:**
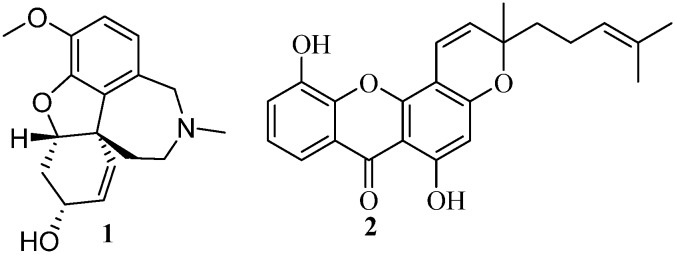
AChE inhibitors.

The alkaloid galantamine (**1**, Figure 1) is used clinically in early stages of AD, nevertheless non-alkaloidal AChE inhibitors are being discovered, including xanthone derivatives [6,7]. Variations in the hydroxylation and methoxylation pattern seem to be important for their activity, as well as extra rings and a hydrophobic side chain as in **2** (Figure 1) [7]. As far as we are aware there are no reports on AChE inhibitors bearing a xanthenedione type structure, but we can find in the literature examples of derivatives that showed DPPH radical scavenging activity [8]. Xanthenedione derivatives are not widespread in Nature but their synthesis still is a hot topic. Several approaches for the synthesis of xanthenediones have been reported, although most of them were strategies towards xanthene-1,8-diones [9]. Xanthene-1,9-diones were less reported [10], but as a part of our ongoing efforts to develop new biologically active compounds we developed a very interesting and efficient strategy towards these type of derivatives [11]. Based on this earlier discovery we became interested in the evaluation of the obtained xanthene-1,9-dione derivatives as antioxidant and acetylcholinesterase inhibitors as well as on their aromatization to xanthone derivatives.

## 2. Results and Discussion

### 2.1. Chemistry

**Scheme 1:**
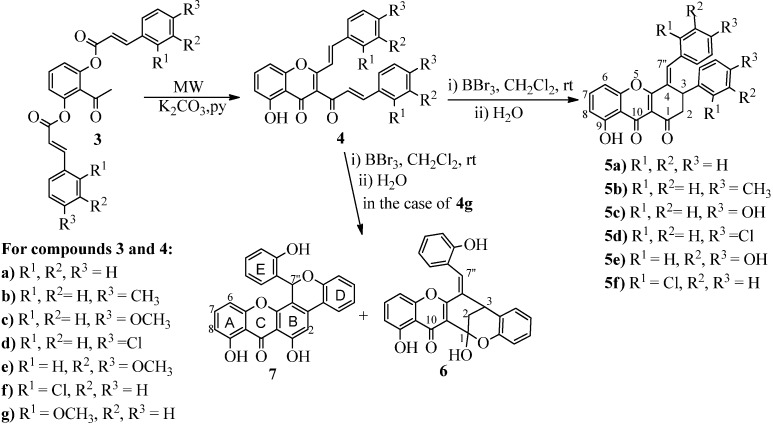
Synthesis of xanthene-1,9(2*H*)-diones.

**Scheme 2:**
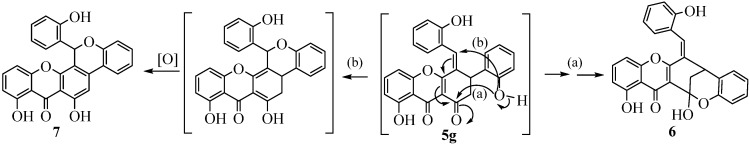
Proposed mechanism.

For the synthesis of xanthene-1,9(2*H*)-dione derivatives a nice portfolio of suitable reactions was developed (Scheme 1). The most important approach was the highly efficient Baker-Venkataraman rearrangement under microwave irradiation of (*E*,*E*)-2-acetyl-1,3-phenylenebis(3-phenylacrylate) derivatives **3** to (*E*,*E*)-3-cinnamoyl-5-hydroxy-2-styrylchromones **4**. Compounds **3** and **4** were previously obtained in good overall yields and were also well characterized [12]. However the application of microwave irradiation is not only a less energetically expensive procedure, but it also presents a very important beneficial effect, the shortening of the reaction time. The cyclization in the presence of the Lewis acid boron tribromide as catalyst of (*E*,*E*)-3-cinnamoyl-5-hydroxy-2-styrylchromones **4** to (*E*)-3-aryl-4-benzylidene-8-hydroxy-3,4 dihydro-1*H-*xanthene-1,9(2*H*)-diones **5** was also very efficient and constitutes an extremely simple synthetic procedure. This protocol works nicely for several derivatives, but its application in the cyclization of (*E*,*E*)-3-cinnamoyl-5-hydroxy-2-styrylchromones **4** bearing electron withdrawing groups is less efficient. The yields in the formation of compounds **5d** and **5f** were lower and more degradation products were observed. Another interesting result was the fact that when (*E*,*E*)-3-cinnamoyl-5-hydroxy-2-styrylchromone (**4g**) was used as starting material, (*E*)-1,13-dihydroxy-6-(2-hydroxybenzylidene)-6,7-dihydro-7,13-methanobenzo[7,8]oxocino [4,3-*b*]chromen-14(13*H*)-one (**6**) was obtained as main product and 6,8-dihydroxy-13-(2-hydroxyphenyl)chromeno[4,3-*c*]xanthen-7(13*H*)-one (**7**) as a by-product (Scheme 1). These results can be explained by an intramolecular cyclization to a hemiketal compound [Scheme 2, path (a) to compound **6**], and other intramolecular cyclization followed by aromatization to the xanthone **7** [Scheme 2, path (b)].

**Scheme 3:**
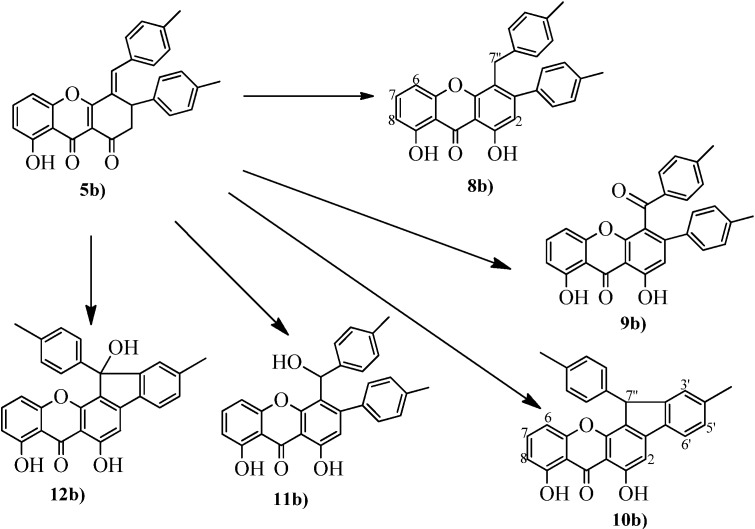
Compounds obtained in the aromatization study of xanthenedione **5b**.

The isolation of xanthone **7** and our ongoing interest in the synthesis of new xanthone derivatives, in particular xanthones bearing aromatic rings attached to the central core [13] prompted us to study the aromatization of xanthene-1,9(2*H*)-diones **5**. (*E*)-8-Hydroxy-4-(4-methylbenzylidene)-3-(4-methylphenyl)-3,4-dihydro-1H*-*xanthene-1,9(2H)-dione (**5b**) was used to find the optimal conditions for the required aromatization. In the first attempts, the reactions with **5b** were performed under acidic conditions to induce the enolization between carbons C-1 and C-2 and the 1,3-shift of H-3 to C-7". Considering that strong or weak acids, at room temperature or heating using classical heating conditions or microwave irradiation did not work, the starting material being recovered, it was decided to attempt basic catalysis. After testing several bases, only DBU and LiHMDS, under the conditions indicated in Table 1 (entry 1 and 2), were able to aromatize the xanthenedione **5b** to the desired xanthone **8b** (Scheme 3). However, as can be understood from Table 1 the results were not good so it was decided to attempt oxidative conditions. Several oxidizing reagents and/or conditions for the oxidation of xanthenedione **5b** were examined, and it was found that although the benzylic carbon is also very reactive the aromatization can be accomplished, and several new xanthone derivatives were isolated (Scheme 3). For instance, xanthone **9b** was obtained in the oxidation of **5b** with DDQ in 1,2,4-trichlorobenzene (TCB) (Table 1, entry 3), although using dry TCB and molecular sieves an interesting cyclization to xanthone **10b** occurred (Table 1, entry 4). Under the same conditions at lower temperature the result was also unexpected (Table 1, entry 5), although it is known that under DDQ oxidation conditions several intramolecular transformations can occur [14]. The oxidation of **5b** was also attempted with the softer oxidizing reagent chloranil, which did not work as well (Table 1, entry 6) and a new xanthone **12b** was obtained (Scheme 3). We tested even milder conditions (Pd/C, Table 1, entry 7) which gave the desired xanthone **8b** in low yields together with xanthones **9b** and **11b**. The results, although were not the expected ones, are interesting. One can conclude that xanthenediones **5** can became useful synthons towards new xanthone derivatives.

**Table 1:** Optimization of the aromatization conditions.

Entry	Catalyst	Experimental conditions ^a^	Obtained compounds ^b^ (%)
1	1.2 equiv DBU	MW (100 °C, 10 min), DMSO	**8b** (18%)
2	LiHMDS	Classical heating (80 °C), toluene	**8b** (<5%)
3	2 equiv DDQ	MW (170 °C, 30 min), TCB	**9b** (28%)
4	1.5 equiv DDQ	MW (170 °C, 30 min), dry TCB, molecular sieves	**10b** (52%)
5	1.3 equiv DDQ	MW (100 °C, 30 min), dry TCB, molecular sieves	**9b** (15%)
			**11b** (30%)
6	2 equiv ChA	MW (170 °C, 30 min), dry TCB, molecular sieves	**9b** (10%)
			**12b** (34%)
7	Pd/C (1:1 w/w)	Classical heating(reflux toluene)	**8b** (15%); **9b** (22%)
			**11b** (25%)

^a^ Only the experimental conditions affording new compounds are described and only the best yields are mentioned; ^b^ Only the major compounds are indicated and starting compound was recovered in all attempts; TCB = 1,2,4-trichlorobenzene.

### 2.2. Nuclear Magnetic Resonance Spectroscopy

The NMR characterization of bis(3-phenylacrylate) derivatives **3**, 3-cinnamoyl-5-hydroxy-2-styrylchromones **4** and xanthene-1,9(2*H*)-diones **5** was previously discussed [11,12] therefore here only the characterization of the new derivatives will be reported.

**Figure 2:**
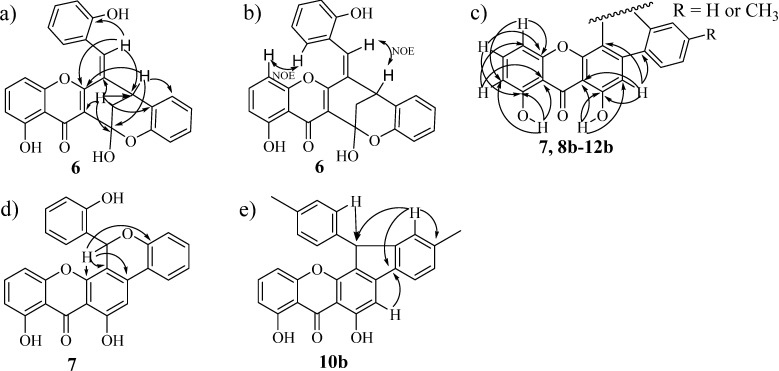
Main HMBC and NOESY correlations.

At the first glances the ^1^H-NMR spectrum of compound **6** seems to indicate the presence of another xanthenedione derivative. But a carefully analysis showed that H-2 protons are equivalent and appear as a doublet at δ 2.56 ppm with a coupling constant (*J* = 3.1 Hz) due to the coupling with proton H-3, which appears as a triplet at δ 4.05 ppm. Another interesting feature is the chemical shift of the H-7'' proton which appears as singlet at δ 7.27 ppm. In the ^13^C-NMR spectrum instead of two carbonyl carbons at δ ~191 and ~180 ppm like in the case xanthenediones just one signal due to a carbonyl carbon resonance is observed at δ 183.2 ppm. These data are considerably different from those of xanthenediones and suggested a different structure. The connectivities found in the HMBC spectrum allowed assigning the quaternary carbon resonances and confirming the assignments of some proton-bearing carbons (Figure 2a). Moreover, the NOESY experiment led to confirm the proposed structure, namely the NOE effects between the signals of H-7'' and H-3 and of H-6 and H-6'' (Figure 2b).

The obtained xanthone derivatives **7** and **8b**–**12b** have in common the central nucleus that presents characteristic ^1^H and ^13^C-NMR spectra. Besides these typical protons and carbon resonances the presence of the aromatic rings A and E can be highlighted by: (i) the singlet due to the H-2 proton resonance at δ 6.79–7.26 ppm; (ii) the two signals due to hydroxyl group proton, as singlets at δ 11.70–11.90 ppm (9-OH) and at δ 11.78–12.22 ppm (1-OH); (iii) the carbonyl carbon resonance at δ 185.3–186.1 ppm; (iv) the connectivities found in the HMBC spectra confirming this structural motif (Figure 2c). The rest of the structures present two types of substitution, xanthones **7**, **10b** and **12b** with an extra ring linking rings B and D and xanthones **8b**, **9b** and **11b** with unlinked substituents at carbons C-3 and C-4. The *p*-tolyl group at carbon C-3 presents the characteristic ^1^H-NMR signals of a *para* substituted aromatic ring and the most distinguishable signals are: (i) the singlet at δ 4.10 ppm, due to the resonance of the benzylic CH_2_ protons of **8b**; (ii) the doublet at δ 6.02 ppm, due to the H-7" proton resonance proton and the broad singlet at δ 2.98 ppm, due to the 7"-hydroxyl group of xanthone **11b**. The main feature of the ^13^C-NMR spectrum of xanthone **9b** is the carbonyl carbon resonance at δ 194.0 ppm. The HMBC connectivities allowed assigning and/or confirming all carbon resonances and confirming the proposed structures as depicted in Scheme 3.

The important signals in the NMR spectra of xanthone **7** are the singlet at δ 7.14 ppm assigned to a proton linked to a carbon that resonates at δ 70.9 ppm and that in the HMBC spectrum showed connectivity with the H-7" proton (Figure 2d). In xanthones **10b** and **12b** the disappearance of an aromatic proton and the consequently change in the NMR pattern of the D ring it is noticeable. In these compounds, it consists in three characteristic signals, a broad singlet or doublet with a *meta* coupling constant due to the H-3' proton (δ ~7.1 ppm), a doublet due to the H-6' proton (δ ~7.6 ppm) and a double doublet assigned to H-5' (δ ~7.2 ppm). The confirmation of the five member ring was provided by the HMBC spectrum (Figure 2e), which showed connectivities between H-3' and C-7" only possible in the proposed structures.

### 2.3. Reducing Power and Radical Scavenging Activity

**Figure 3:**
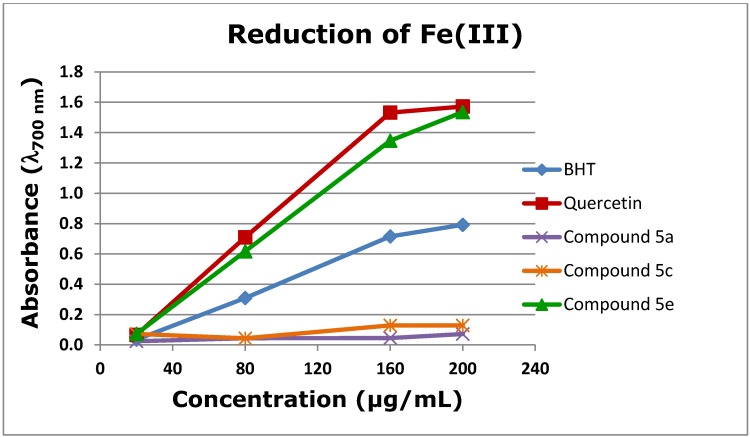
Reducing activity of xanthenediones **5c**,**e**.

As was raised in the Introduction there are several diseases and symptoms directly related to oxidative stress, such as: atherosclerosis, Alzheimer’s, cancer, the aging process and central nervous system degeneration [15]. Antioxidant compounds can become important in preventing and/or treating these diseases. In order to evaluate the antioxidant activity of xanthenediones **5**, we tested their ability to scavenge DPPH radicals and to reduce iron(III) and the results are presented in Figure 3 and Table 2.

**Table 2:** Radical scavenging activity and acetylcholinesterase inhibitory effect of xanthenediones (**5**).

Compounds	Radical Scavenging Activity		Anti-AChE IC_50_^c^ (µM ± SD, *n =* 4)
	EC_50_^a^ (µM ± SD)	AE ^b^	
**5a**	nd	nd	>381
**5b**	nd	nd	>355
**5c**	>552	nd	41.1 ± 6.1
**5d**	nd	nd	>325
**5e**	3.79 ± 0.06	0.026	31.0 ± 0.09
**5f**	nd	nd	>325
**Trolox**	18.7 ± 0.65	0.011	-
**Quercetin**	5.97 ± 0.11	0.0042	-
**Galantamine**	-	-	211.8 ± 9.5

^a^ The required concentration of the compound to reduce the DPPH^•^ concentration to 50%; ^b^ The antiradical efficiency (AE = 1/(EC_50_ × T_EC50_); T_EC50_ the time required to reach the steady state for the EC_50_; ^c^ The required concentration of the compound to inhibit 50% of acetylcholinesterase activity; nd not determined.

Reducing power is the manifestation of the electron donating capacity of a compound and can be associated with its antioxidant activity [16] and also with structural features such as *ortho*-dihydroxyl groups or hydroxyl groups that can form a hydrogen bond with a carbonyl group. Thus, xanthenediones **5a**, **5c** and **5e** were tested using the potassium ferricyanide reduction method [17]. BHT and quercetin were used as references due to their well-known antioxidant properties. Quercetin has an additional significance, as it is structurally more similar to the tested compounds. The compounds were tested at four concentrations, and naturally the reducing ability increased with concentration (Figure 3). Xanthenediones **5a** and **5c** did not significantly reduce ferric ion. Xanthenedione **5e** was able not only to reduce considerably the ferric ion, but was also more efficient than BHT and similar to quercetin (Figure 3). These results confirm that a catechol moiety in a molecule is important for the reducing power activity. Considering the above mentioned conclusions and the fact that xanthenedione **5c** did not violate any of the Lipinski’s ‘rule of five’ (Table 3) we decided to test also its radical scavenging activity (Table 2). However the solubility of xanthenedione **5c** did not allow an EC_50_ calculation. At the maximum concentration at which xanthenedione **5c** is soluble (552 µM), the DPPH quenched was just 36%.

### 2.4. Acetylcholinesterase Inhibitory Activity (Anti-AChE)

Acetylcholinesterase (AChE) plays a very important role in nerve transmission, as an adhesion protein, as a bone matrix protein, in neurite growth and in the production of amyloid fibrils, found in the brain cells of patients with Alzheimer’s disease [6]. Inhibition of AChE will, therefore, allow the stimulatory activity of acetylcholine (ACh), which appears to be associated with cognitive processes, memory, myasthenia gravis, and glaucoma and as an antidote to various toxins [6]. From the literature [7], it is known that the xanthone ring itself did not appear to automatically confer activity; in fact the substitution pattern has a marked influence on the activity level. Since the prepared xanthenediones **5** are structurally related to xanthones they were evaluated for their anti-AChE activity (Table 2). The results showed that two derivatives are even more active than the control used, the known active compound galantamine (Table 2), which is a pure competitive inhibitor of AChE used clinically in early stages of Alzheimer’s disease. Both active compounds present more than one hydroxyl group in their structure, which suggests that the presence of hydroxyl groups in the 3-aryl and 4-benzylidene moieties is essential for the activity. Although there are a few differences in the AChE inhibition assays carried out by different authors, it is possible to compare our results with those previously reported for xanthones. The two active compounds in the present work are much stronger inhibitors than those isolated from the fungus *Amauroderma amoiensis*, where the highest inhibition of the enzyme at 50 µM was only 46.3% [18]. The inhibition potencies of xanthenediones **5c** and **5e** is higher or of the same order of magnitude to those reported by a study comparing inhibition potency and inhibition types of six different xanthones [7]. Furthermore xanthenedione **5c** has a mixed type inhibition of AChE, producing a combination of partially competitive and non-competitive inhibition while xanthenedione **5e** shows an almost pure competitive type inhibition. Compounds with competitive or mixed inhibition of AChE are of great interest in Alzheimer’s disease therapy. Dual binding site inhibitors, which interact both with the catalytic and the peripheral anionic sites of the enzyme are particularly interesting, since they not only alleviate cognitive deficits but also behave as disease-modifying agents by inhibiting the β-amyloid peptide aggregation [19].

Molecular properties such as the molecular weight, hydrophobicity (LogP), and the total polar surface area (TPSA) have been used extensively in modern drug discovery because they affect drug absorption, bioavailability, metabolism, toxicity as well as the hydrophobic drug-receptor interactions [20]. These and other parameters related with drug-likeness properties of xanthenediones **5** are listed in Table 3 [21].

**Table 3:** Drug-likeness property/Lipinski’s ‘rule of five’ parameters calculated for xanthenediones (**5**) **^a^**.

Compound	Molecular Weight	miLog*P*	*n*-ROTB	*n*-ON Acceptors	*n*-OHNH Donors	*n* Violations	TPSA
**5a**	394.426	5.503	2	4	1	1	67.51
**5b**	422.480	6.4	2	4	1	1	67.51
**5c**	426.424	4.544	2	6	3	0	107.96
**5d**	463.316	6.859	2	4	1	1	67.51
**5e**	458.422	3.566	2	8	5	0	148.42
**5f**	463.316	6.11	2	4	1	1	67.51

^a^ log *P*, octanol-water partition coefficients; *n*-ROTB, number of rotatable bonds; *n*-ON, number of hydrogen acceptors; *n*-OHNH, number of hydrogen bond donors; *n* violations, number of violations according to the Lipinski ‘rule of five’; TPSA, topological polar surface area.

It is interesting to highlight that the xanthenediones **5e** and **5c**, highly active in the biological assays, present zero violations of Lipinski’s ‘rule of five’. On the other hand, since the TPSA parameter can be correlated with human intestinal absorption, the Caco-2 monolayers permeability, and blood-brain barrier penetration, drugs with TPSA lower than 140 Å^2^ and lower molecular flexibility (n-ROTB ≤ 6) contribute to higher intestinal permeability and higher human dose absorption [22,23]. Therefore, we can conclude that xanthenedione **5c** combines higher AChE activity with good oral bioavailability properties.

## 3. Experimental

### 3.1. General Information

The ^1^H- and ^13^C-NMR, HSQC and HMBC spectra were measured in CDCl_3_ on Bruker Avance 300 and 500 spectrometers (300.13 MHz and 500.13 for ^1^H and 75.47 and 125.75 MHz for ^13^C, respectively), using TMS as internal standard. The low-pass *J*-filter portion of the HMBC experiment was optimized for an average of one-bond heteronuclear coupling of 145 Hz; the delay for evolution of long-range couplings was optimized for 7 and 2 Hz. Chemical shifts were reported in δ units (ppm) and coupling constants (*J*) in Hz. The MS spectra were obtained using ESI positive mode with a Q-TOF 2 mass spectrometer. High resolution mass spectra (HRMS-ESI+) were performed on a microTOF (focus) mass spectrometer. Ions were generated using an Apollo II (ESI) source. Ionization was achieved by electrospray, using a voltage of 4500 V applied to the needle, and a counter voltage between 100 and 150 V applied to the capillary. Reactions were routinely monitored by thin-layer chromatography (TLC) in silica gel 60 (Merck F_245_ plates) and the products visualized with ultraviolet lamp (254 and/or 365 nm). The chromatographic purifications were carried out by CC on silica gel 60 (70–230 mesh) and prep. TLC on silica gel (Merck silica gel 60 F_254_), being the spots visualized under a UV lamp (at 254 and/or 366 nm).

### 3.2. Synthesis

Compounds **3** and **4** were synthesised according to previously reported methodologies and the obtained NMR data were also identical to the data previously reported in the literature [11,12]. In the case of xanthenediones **5**, although the synthetic methodology was previously reported only the characterization of derivatives **5c** and **5d** had been published [11].

*(E)-3-Aryl-4-benzylidene-8-hydroxy-3,4-dihydro-1H-xanthene-1,9(2H)-dione* (**5a**). This compound was obtained by the reaction of (*E*,*E*)-3-cinnamoyl-5-hydroxy-2-styrylchromone (**4a**, 157.8 mg, 0.400 mmol) with BBr_3_ (2 mL of a 1 mol L^−^^1^ solution in CH_2_Cl_2_). After the slowly addition of BBr_3_ the mixture was stirred at room temp. for 3 h, then water (80 mL) was added. The resulting reaction mixture was stirred for 4 h and extracted with CH_2_Cl_2_ (3 × 80 mL). The combined extracts were evaporated and purified by TLC using CH_2_Cl_2_ as eluent. The desired compound **5a** was obtained as yellow crystals (107.3 mg, 68%). M.p. 216–218 °C. ^1^H-NMR (300 MHz, CDCl_3_, TMS) δ (ppm): 3.08 (dd, 1H, *J* = 3.2 and 15.5 Hz, H-2_trans_); 3.15 (dd, 1H, *J* = 5.0 and 15.5 Hz, H-2*_cis_*); 4.76 (dd, 1H, *J* = 3.2 and 5.0 Hz, H-3); 6.82 (dd, 1H, *J* = 0.7 and 8.4 Hz, H-8); 7.02 (dd, 1H, *J* = 0.7 and 8.4 Hz, H-6); 7.24–7.30 (m, 3H, H-2',6' and H-4'); 7.31–7.36 (m, 2H, H-3',5'); 7.38 (br s, 5H, H-2",6", H-3",5" and H-4"); 7.57 (t, 1H, *J* = 8.4 Hz, H-7); 8.11 (s, 1H, H-7"); 12.63 (s, 1H, 9-OH). ^13^C-NMR (75 MHz, CDCl_3_, TMS) δ (ppm): 40.1 (C-3); 45.6 (C-2); 106.8 (C-6); 111.0 (C-9a); 112.7 (C-8); 113.4 (C-10a); 126.9 (C-2' and C-6'); 127.5 (C-4'); 128.9 (C-3" and C-5"); 129.2 (C-3' and C-5'); 129.5 (C-2" and C-6"); 129.7 (C-4); 129.9 (C-4"); 134.2 (C-1"); 136.0 (C-7); 139.0 (C-7"); 139.9 (C-1'); 154.7 (C-5a); 161.7 (C-9); 169.4 (C-4a); 180.1 (C-10); 191.3 (C-1). ESI-MS *m/z* 395 [M+H]^+^; 417 [M+Na]^+^; 433 [M+K]^+^. HR-ESIMS (positive ion): *m/z* calcd. for C_26_H_19_O_4_ [M+H]^+^, 395.1278; found, 395.1275.

*(E)-8-Hydroxy-4-(4-methylbenzylidene)-3-(4-methylphenyl)-3,4-dihydro-1H-xanthene-1,9(2H)-dione* (**5b**). This compound was obtained by the reaction of (*E*,*E*)-3-cinnamoyl-5-hydroxy-2-styrylchromone (**4b**, 172.8 mg, 0.409 mmol) with BBr_3_ (2 mL of a 1 mol L solution in CH_2_Cl_2_) and following the above described procedure. The desired compound **5b** was obtained as yellow crystals (153.8 mg, 89%). M.p. 201–202 °C. ^1^H-NMR (300 MHz, CDCl_3_, TMS) δ (ppm): 2.29 (s, 3H, 4'-CH_3_); 2.37 (s, 3H, 4"-CH_3_); 3.05 (dd, 1H, *J* = 3.1 and 15.5 Hz, H-2_trans_); 3.12 (dd, 1H, *J* = 5.1 and 15.5 Hz, H-2*_cis_*); 4.74–4.76 (m, 1H, H-3); 6.82 (dd, 1H, *J* = 0.8 and 8.3 Hz, H-8); 7.02 (dd, 1H, *J* = 0.8 and 8.3 Hz, H-6); 7.10 (d, 2H, *J* = 8.0 Hz, H-3',5'); 7.13 (d, 1H, *J* = 8.0 Hz, H-2',6'); 7.18 (d, 2H, *J* = 8.1 Hz, H-3",5"); 7.29 (d, 2H, *J* = 8.1 Hz, H-2",6"); 7.56 (t, 1H, *J* = 8.3 Hz, H-7); 8.07 (s, 1H, H-7"); 12.67 (s, 1H, 9-OH). ^13^C-NMR (75 MHz, CDCl_3_, TMS) δ (ppm): 20.9 (4'-CH_3_); 21.4 (4"-CH_3_); 39.9 (C-3); 45.6 (C-2); 106.8 (C-6); 111.0 (C-9a); 112.6 (C-8); 113.3 (C-10a); 126.8 (C-2' and C-6'); 129.0 (C-4); 129.6 (C-3" and C-5"); 129.7 (C-3' and C-5'); 129.9 (C-2" and C-6"); 131.4 (C-1"); 135.9 (C-7); 136.8 (C-1'); 137.1 (C-4'); 139.0 (C-7"); 140.5 (C-4"); 154.7 (C-5a); 161.7 (C-9); 169.7 (C-4a); 180.2 (C-10); 191.5 (C-1). ESI-MS *m/z* 423 [M+H]^+^; 445 [M+Na]^+^. HR-ESIMS (positive ion): *m/z* calcd. for C_28_H_22_O_4_ [M]^+^, 423.1591; found, 423.1587.

*(E)-8-Hydroxy-4-(3,4-dihydroxybenzylidene)-3-(3,4-dihydroxyphenyl)-3,4-dihydro-1H-xanthene-1,9-(2H)-dione* (**5e**). This compound was obtained by the reaction of (*E*,*E*)-3-cinnamoyl-5-hydroxy-2-styrylchromone (**4e**, 205.8 mg, 0.400 mmol) with BBr_3_ (2 mL of a 1 mol L^−^^1^ solution in CH_2_Cl_2_) and following the above described procedure. The desired compound **5e** was filtered off and obtained as orange crystals (183.2 mg, 89%). M.p. 275–281 °C. ^1^H-NMR (500 MHz, DMSO-d_6_, TMS) δ (ppm): 2.68 (dd, 1H, *J* = 2.7 and 14.8 Hz, H-2_trans_); 3.16 (dd, 1H, *J* = 5.7 and 14.8 Hz, H-2*_cis_*); 4.60 (dbr, 1H, *J* = 2.7 Hz, H-3); 6.57 (dd, 1H, *J* = 2.3 and 8.2 Hz, H-6'); 6.67 (d, 1H, *J* = 2.3 Hz, H-2'); 6.67 (d, 1H, *J* = 8.2 Hz, H-5');6.77 (d, 1H, *J* = 8.3 Hz, H-5"); 6.83 (d, 1H, *J* = 8.3 Hz, H-8); 6.87 (dd, 1H, *J* = 1.8 and 8.3 Hz, H-6"); 6.97 (d, 1H, *J* = 1.8 Hz, H-2"); 7.29 (d, 1H, *J* = 8.3 Hz, H-6); 7.71 (t, 1H, *J* = 8.3 Hz, H-7); 8.05 (s, 1H, H-7"); 8.87 (s, 1H, 4'-OH); 8.91 (s, 1H, 3'-OH); 9.19 (s, 1H, 3"-OH); 9.84 (s, 1H, 4"-OH); 12.77 (s, 1H, 9-OH). ^13^C-NMR (75 MHz, DMSO-d_6_, TMS) δ (ppm): 39.3 (C-3); 46.2 (C-2); 107.8 (C-6); 110.3 (C-9a); 111.9 (C-8); 112.4 (C-10a); 114.3 (C-2'); 115.8 (C-5'); 116.1 (C-5"); 117.9 (C-6' and C-2"); 123.5 (C-6"); 125.9 (C-4*); 126.0 (C-1"*); 131.4 (C-1'); 136.4 (C-7); 139.4 (C-7"); 144.3 (C-3'); 145.4 (C-4'**); 145.5 (C-3"**); 148.3 (C-4"); 154.5 (C-5a); 160.7 (C-9); 169.9 (C-4a); 179.7 (C-10); 191.4 (C-1). *It may be interchanged. **It may be interchanged. ESI-MS *m/z* 459 [M+H]^+^; 481 [M+Na]^+^. HR-ESIMS (positive ion): *m/z* calcd. for C_26_H_18_O_8_ [M]^+^, 458.1002; found, 458.1003.

*(E)-4-(2-Chlorobenzylidene)-3-(2-chlorophenyl)-8-hydroxy-3,4-dihydro-1H-xanthene-1,9(2H)-dione* (**5f**). This compound was obtained by the reaction of (*E*,*E*)-3-cinnamoyl-5-hydroxy-2-styrylchromone **4f** (185.8 mg, 0.401 mmol) with BBr_3_ (2 mL of a 1 mol L^−^^1^ solution in CH_2_Cl_2_) and following the above described procedure. The desired compound **5f** was obtained as yellow crystals (100.3 mg, 54%). D.p. 242 °C. ^1^H-NMR (300 MHz, CDCl_3_, TMS) δ (ppm): 2.97 (dd, 1H, *J* = 2.2 and 15.4 Hz, H-2_trans_); 3.11 (dd, 1H, *J* = 6.6 and 15.4 Hz, H-2*_cis_*); 4.91 (dd, 1H, *J* = 2.2 and 6.6 Hz, H-3); 6.82 (br d, 1H, *J* = 7.8 Hz, H-6"); 6.89 (br d, 1H, *J* = 8.2 Hz, H-8); 7.09 (br d, 1H, *J* = 8.2 Hz, H-6); 7.10–7.14 (m, 2H, H-5' and H-5"); 7.14–7.18 (m, 1H, H-6'); 7.23 (ddd, 1H, *J* = 2.1, 7.1 and 7.6 Hz, H-4'); 7.32 (ddd, 1H, *J* = 1.3, 7.4 and 8.1 Hz, H-4"); 7.42 (dd, 1H, *J* = 1.0 and 7.6 Hz, H-3'); 7.48 (dd, 1H, *J* = 0.6 and 8.1 Hz, H-3"); 7.63 (t, 1H, *J* = 8.2 Hz, H-7); 8.26 (s, 1H, H-7"); 12.59 (s, 1H, 9-OH). ^13^C-NMR (75 MHz, CDCl_3_, TMS) δ (ppm): 38.3 (C-3); 43.9 (C-2); 107.0 (C-6); 111.2 (C-9a); 113.0 (C-8); 114.1 (C-10a); 126.9 (C-6'); 127.6 (C-5' *); 127.7 (C-5" *); 129.0 (C-6"); 129.1 (C-4'); 130.0 (C-3"); 130.2 (C-4); 130.7 (C-3'); 131.0 (C-4"); 132.5 (C-1"); 132.9 (C-2'); 135.0 (C-2"); 136.3 (C-7); 136.5 (C-7"); 138.7 (C-1'); 154.8 (C-5a); 161.8 (C-9); 168.7 (C-4a); 180.1 (C-10); 191.3 (C-1). * May be interchanged. ESI-MS *m/z* 463 ([M+H]^+^, ^35^Cl); 465 ([M+H]^+^, ^35^Cl, ^37^Cl); 485 ([M+Na]^+^, ^35^Cl); 487 ([M+Na]^+^, ^35^Cl, ^37^Cl); 489 ([M+Na]^+^, ^37^Cl). HR-ESIMS (positive ion): *m/z* calcd. for C_26_H_16_^35^Cl_2_O_4_ ([M]^+^, ^35^Cl), 462.0426; found, 462.0406. Calcd. for C_26_H_16_^35^Cl^37^ClO_4_ ([M]^+^, ^35^Cl^37^Cl), 464.0396; found, 464.0374. Calcd. for C_26_H_16_^37^Cl_2_O_4_ ([M]^+^, ^37^Cl), 466.0367; found, 466.0384.

*(E)-1,13-Dihydroxy-6-(2-hydroxybenzylidene)-6,7-dihydro-7,13-methanobenzo[7,8]oxocino[4,3-b]-chromen-14(13H)-one* (**6**). This compound was obtained by the reaction of (*E*,*E*)-3-cinnamoyl-5-hydroxy-2-styrylchromone (**4g**, 181.8 mg, 0.400 mmol) with BBr_3_ (2 mL of a 1 mol L^−^^1^ solution in CH_2_Cl_2_). After the slowly addition of BBr_3_ the mixture was stirred at room temp. for 3 h, then water (80 mL) was added. The resulting reaction mixture was stirred for 4h and extracted with CH_2_Cl_2_ (3 × 80 mL). The combined extracts were evaporated and purified by TLC using CH_2_Cl_2_ as eluent. The desired compound **6** was obtained as orange crystals (74.5 mg, 41%). M.p. 195–198 °C. ^1^H-NMR (300 MHz, CDCl_3_, TMS) δ (ppm): 2.56 (d, 2H, *J* = 3.1 Hz, H-2); 4.05 (t, 1H, *J* = 3.1 Hz, H-3); 6.57 (dd, 1H, *J* = 0.8 and 8.1 Hz, H-6); 6.64 (dd, 1H, *J* = 0.8 and 8.1 Hz, H-8); 6.79 (br d, 1H, *J* = 8.0 Hz, H-3'); 6.85 (ddd, 1H, *J* = 1.1, 7.5 and 8.1 Hz, H-5'); 7.07–7.11 (m, 1H, H-4'); 7.12 (br d, 1H, *J* = 7.5 Hz, H-6'); 7.18–7.23 (m, 1H, H-5"); 7.27 (s, 1H, H-7"); 7.34 (dd, 1H, *J* = 1.1, 7.6 Hz, H-6"); 7.37 (t, 1H, *J* = 8.1 Hz, H-7); 7.37–7.40 (m, H, H-3"); 7.42 (m, 1H, H-4"); 13.00 (s, 1H, 9-OH). ^13^C-NMR (75 MHz, CDCl_3_, TMS) δ (ppm): 31.3 (C-2); 38.3 (C-3); 100.7 (C-1); 104.9 (C-10a); 107.1 (C-6); 108.5 (C-9a); 111.0 (C-8); 116.9 (C-3"); 118.1 (C-3'); 120.2 (C-1"); 121.4 (C-5'); 122.4 (C-1'); 124.9 (C-5"); 127.1 (C-6"); 128.0 (C-6'); 128.7 (C-4'); 129.1 (C-7"); 131.4 (C-4); 132.5 (C-4); 136.5 (C-7); 151.4 (C-2"); 152.1 (C-2'); 156.0 (C-5a); 158.6 (4a); 162.8 (C-9); 183.2 (C-10). ESI-MS *m/z* 409 [M−H_2_O+H]^+^; 431 [M−H_2_O+Na]^+^; 447 [M−H_2_O+Na]^+^. HR-ESIMS (positive ion): *m/z* calcd. for C_26_H_19_O_6_ [M+H]^+^, 427.1176; found, 427.1181; Calcd. for C_26_H_17_O_5_ [M-H_2_O+H]^+^, 409, 1070; found, 409.1072.

*6,8-Dihydroxy-13-(2-hydroxyphenyl)chromeno[4,3-c]xanthen-7(13H)-one* (**7**). This compound was obtained following the above described procedure for compound **6**. The desired compound **7** was obtained as orange crystals (5.5 mg, 3%). D.p. 242 °C. ^1^H-NMR (300 MHz, CDCl_3_, TMS) δ (ppm): 6.43 (d, 1H, *J* = 1.2 and 7.9 Hz, H-6"); 6.61 (dt, 1H, *J* = 1.1 and 7.9 Hz, H-5"); 6.82 (dd, 2H, *J* = 0.8 and 8.3 Hz, H-8 and H-6); 6.94 (dd, 1H, *J* = 1.0 and 8.1 Hz, H-3"); 7.01 (dd, 1H, *J* = 1.1 and 8.1 Hz, H-3'); 7.08 (dt, 1H, *J* = 1.1 and 7.7 Hz, H-5'); 7.14 (s, 1H, H-7"); 7.14–7.17 (m, 1H, H-4"); 7.26 (s, 1H, H-2); 7.25–7.30 (m, 1H, H-4'); 7.57 (t, 1H, *J* = 8.3 Hz, H-7); 7.74 (dd, 1H, *J* = 1.5 and 7.7 Hz, H-6'); 11.80 (s, 1H, 9-OH); 12.01 (s, 1H, 1-OH). ^13^C-NMR (75 MHz, CDCl_3_, TMS) δ (ppm): 70.9 (C-7"); 104.9 (C-2); 106.7 (C-10a); 107.4 (C-6); 107.9 (C-9a); 108.9 (C-4); 111.4 (C-8); 116.8 (C-3"); 118.0 (C-3'); 120.2 (C-5"); 121.3 (C-1'); 123.3 (C-5' and C-1"); 124.7 (C-6'); 127.8 (C-6"); 130.5 (C-4"); 132.1 (C-4'); 137.6 (C-7); 139.2 (C-3); 152.0 (C-2'); 152.2 (C-4a); 155.7 (C-2"); 155.9 (C-5a); 161.4 (C-9); 161.7 (C-1); 185.5 (C-10). ESI-MS *m/z* 425 [M+H]^+^; 447 [M+Na]^+^; 463 [M+K]^+^.

*1,8-Dihydroxy-4-(4-methylbenzyl)-3-(4-methylphenyl)-9H-xanthen-9-one* (**8b**). This compound was obtained by the treatment of a solution of (*E*)-8-hydroxy-4-(4-methylbenzylidene)-3-(4-methylphenyl)-3,4-dihydro-1*H-*xanthene-1,9(2*H*)-dione (**5b**, 56.7 mg, 0.13 mmol) in DMSO (10 mL) with DBU (1.2 equiv, 24 µL, 0.16 mmol). The mixture was then irradiated in an Ethos SYNTH microwave (Milestone Inc., Sorisole, Italy) at 100 °C for 10 min. After this period the reaction mixture was poured onto ice (10 g) and the pH was adjusted to 3–4 with diluted HCl and extracted with chloroform. The desired compound **8b** was obtained by TLC purification using a mixture of hexane/CH_2_Cl_2_ (3:1) as eluent, as orange crystals, 18% yield (10.2 mg). M.p. 155–157 °C; ^1^H-NMR (300 MHz, CDCl_3_, TMS) δ (ppm): 2.26 (s, 3H, 4"-CH_3_); 2.40 (s, 3H, 4'-CH_3_); 4.10 (s, 2H, H-7"); 6.77 (dd, 1H, *J* = 0.7 and 8.8 Hz, H-8); 6.79 (s, 1H, H-2); 6.81 (dd, 1H, *J* = 0.7 and 8.8 Hz, H-6); 6.90 (d, 2H, *J* = 8.1 Hz, H-2",6"); 6.99 (d, 2H, *J* = 8.1 Hz, H-3",5"); 7.17–7.19 (m, 2H, H-2',6'); 7.19–7.21 (m, 2H, H-3',5'); 7.55 (t, 1H, *J* = 8.8 Hz, H-7); 11.78 (s, 1H, 1-OH); 11.86 (s, 1H, 9-OH). ^13^C-NMR (75 MHz, CDCl_3_, TMS) δ (ppm): 20.9 (4"-CH_3_); 21.2 (4'-CH_3_); 31.7 (C-7"); 107.0 (C-10a); 107.2 (C-6); 107.7 (C-9a); 110.7 (C-8); 112.5 (C-2); 117.1 (C-4*); 127.8 (C-2" and C-6"); 128.6 (C-2' and C-6'); 128.9 (C-3', C-3", C-5' and C-5"); 135.2 (C-4"); 137.1 (C-1') 137.3 (C-7); 137.4 (C-3*); 137.7 (C-1"); 137.9 (C-4'); 152.3 (C-4a); 156.3 (C-5a); 158.8 (C-1); 161.2 (C-9); 186.1 (C-10). * May be interchanged. ESI-MS *m/z* 423 [M+H]^+^; 445 [M+Na]^+^. HR-ESIMS (positive ion): *m/z* calcd. for C_28_H_22_O_4_ [M]^+^, 422.1518; found, 422.1520.

*1,8-Dihydroxy-4-(4-methylbenzoyl)-3-(4-methylphenyl)-9H-xanthen-9-one* (**9b**). This compound was obtained by the treatment of a solution of (*E*)-8-hydroxy-4-(4-methylbenzylidene)-3-(4-methylphenyl)-3,4-dihydro-1*H-*xanthene-1,9(2*H*)-dione (**5b**, 66.9 mg, 0.16 mmol) in 1,2,4-trichlorobenzene (TCB) (10 mL) with DQQ (2.0 equiv, 72.0 mg, 0.32 mmol). The mixture was then irradiated in an Ethos SYNTH microwave (Milestone Inc.) at 170 °C for 30 min. After this the TCB was removed by column chromatography using hexane as eluent and the reaction mixture was eluted with CH_2_Cl_2_. The desired compound **9b** was obtained by TLC purification using successively hexane and CH_2_Cl_2_ as eluents, as yellow solid, 28% yield (19.1 mg). M.p. 180–183 °C; ^1^H-NMR (300 MHz, CDCl_3_, TMS) δ (ppm): 2.28 (s, 3H, 4'-CH_3_); 2.36 (s, 3H, 4"-CH_3_); 2.98 (sb, 1H, 7"-OH); 6.02 (d, 1H, *J* = 9.0 Hz, H-7"); 6.60 (dd, 1H, *J* = 0.8 and 8.4 Hz, H-6); 6.77 (dd, 1H, *J* = 0.8 and 8.4 Hz, H-8); 6.91 (s, 1H, H-2); 7.06 (d, 2H, *J* = 8.2 Hz, H-3',5'); 7.15 (d, 2H, *J* = 8.2 Hz, H-3",5"); 7.22 (d, 2H, *J* = 8.2 Hz, H-2',6'); 7.50 (t, 1H, *J* = 8.4 Hz, H-7); 7.65 '(d, 2H, *J* = 8.2 Hz, H-2",6"); 11.71 (s, 1H, 9-OH); 12.06 (s, 1H, 1-OH). ^13^C-NMR (75 MHz, CDCl_3_, TMS) δ (ppm): 21.2 (4'-CH_3_); 21.7 (4"-CH_3_); 106.1 (C-10a); 107.4 (C-6); 107.7 (C-9a); 111.1 (C-8); 112.3 (C-2); 118.6 (C-4); 128.5 (C-2' and C-6'); 129.2 (C-3', C-5', C-3" and C-5"); 129.6 (C-2" and C-6"); 135.4 (C-1"); 135.6 (C-1'); 137.6 (C-7); 138.7 (C-4'); 144.4 (C-4"); 150.4 (C-3); 153.4 (C-4a); 155.9 (C-5a); 161.2 (C-9); 161.4 (C-1); 185.7 (C-10); 194.0 (C-7");. ESI-MS *m/z* 437 [M+H]^+^, 459 [M+Na]^+^, 475 [M+K]^+^. HR-ESIMS (positive ion): *m/z* calcd. for C_28_H_20_O_5_ [M]^+^, 436,1311; found, 436.1319.

*6,8-Dihydroxy-2-methyl-13-(4-methylphenyl)indeno[1,2-c]xanthen-7(13H)-one* (**10b**). This compound was obtained by the treatment of a solution of (E)-8-hydroxy-4-(4-methylbenzylidene)-3-(4-methylphenyl)-3,4-dihydro-1*H-*xanthene-1,9(2*H*)-dione (**5b**, 50.5 mg, 0.12 mmol) in dry 1,2,4-trichlorobenzene (TCB) (10 mL) and in the presence of 100 mg molecular sieves with DQQ (1.5 equiv, 40.6 mg, 0.18 mmol). The mixture was then irradiated in an Ethos SYNTH microwave (Milestone Inc.) at 170 °C for 30 min. After this the TCB was removed by column chromatography using hexane as eluent and the reaction mixture was eluted with CH_2_Cl_2_. The desired compound **10b** was obtained by TLC purification using a mixture of hexane/CH_2_Cl_2_ (3:2) as eluent, as yellow crystals, 52% yield (26.0 mg). D.p. 195 °C; ^1^H-NMR (300 MHz, CDCl_3_, TMS) δ (ppm): 2.28 (s, 3H, 4"-CH_3_); 2.36 (s, 3H, 4'-CH_3_); 5.14 (s, 1H, H-7"); 6.64 (dd, 1H, *J* = 0.7 and 8.3 Hz, H-6); 6.70 (dd, 1H, *J* = 0.7 and 8.3 Hz, H-8); 7.01 (d, 2H, *J* = 8.2 Hz, H-2",6"); 7.06 (d, 2H, *J* = 8.2 Hz, H-3",5"); 7.13 (br s, 1H, H-3'); 7.14 (s, 1H, H-2); 7.20 (br d, 1H, *J* = 7.9 Hz, H-5'); 7.47 (t, 1H, *J* = 8.3 Hz, H-7); 7.67 (d, 1H, *J* = 7.9 Hz, H-6'); 11.90 (s, 1H, 9-OH); 12.05 (s, 1H, 1-OH). ^13^C-NMR (75 MHz, CDCl_3_, TMS) δ (ppm): 21.1 (4"-CH_3_); 21.8 (4'-CH_3_); 51.5 (C-7"); 102.1 (C-2); 106.4 (C-10a); 106.9 (C-6); 107.8 (C-9a); 110.7 (C-8); 121.1 (C-6'); 123.3 (C-4); 126.1 (C-3'); 128.0 (C-2" and C-6"); 128.6 (C- 5'); 129.2 (C-3" and C-5"); 136.4 (C-4"); 136.8 (C-1"*); 136.9 (C-7); 137.0 (C-1'*); 140.3 (C-4'); 150.7 (C-2'); 151.4 (C-4a); 152.0 (C-3); 155.9 (C-5a); 161.3 (C-9); 162.2 (C-1); 185.5 (C-10) * May be interchanged. ESI-MS *m/z* 421 [M+H]^+^; 443 [M+Na]^+^, 459 [M+K]^+^. HR-ESIMS (positive ion): *m/z* calcd. for C_28_H_20_O_4_ [M]^+^, 420.1362; found, 420.1364.

*1,8-Dihydroxy-4-[(4-methylphenyl)hydroxymethyl]-3-(4-methylphenyl)-9H-xanthen-9-one* (**11b**). This compound was obtained by the treatment of a solution of (*E*)-8-hydroxy-4-(4-methylbenzylidene)-3-(4-methylphenyl)-3,4-dihydro-1*H-*xanthene-1,9(2*H*)-dione (**5b**, 58.8 mg, 0.14 mmol) in dried 1,2,4-trichlorobenzene (TCB) (10 mL) and in the presence of 100 mg molecular sieves with DQQ (1.3 equiv, 42.0 mg, 0.18 mmol). The mixture was then irradiated in an Ethos SYNTH microwave (Milestone Inc.) at 100 °C for 30 min. After this the TCB was removed by column chromatography using hexane as eluent and the reaction mixture was eluted with CH_2_Cl_2_. The desired compound **11b** was obtained by TLC purification using a mixture of hexane/CH2Cl2 (3:2) as eluent, as yellow crystals, 30% yield (18.6 mg). M.p. 134–136 °C; ^1^H-NMR (300 MHz, CDCl_3_, TMS) δ (ppm): 2.29 (s, 3H, 4"-CH_3_); 2.39 (s, 3H, 4'-CH_3_); 6.02 (d, 1H, *J* = 9.0 Hz, H-7"); 6.55 (br d, 1H, *J* = 8.2 Hz, H-6); 6.74 (dd, 1H, *J* = 0.7 and 8.2 Hz, H-8); 6.79 (s, 1H, H-2); 7.07 (d, 2H, *J* = 8.0 Hz, H-3",5"); 7.17 (d, 2H, *J* = 8.0 Hz, H-2",6"); 7.22 (d, 2H, *J* = 8.1 Hz, H-3',5'); 7.28 (d, 2H, *J* = 8.1 Hz, H-2',6'); 7.48 (t, 1H, *J* = 8.2 Hz, H-7); 11.70 (s, 1H, 9-OH); 11.84 (s, 1H, 1-OH). ^13^C-NMR (75 MHz, CDCl_3_, TMS) δ (ppm): 21.0 (4"-CH_3_); 21.2 (4'-CH_3_); 70.8 (C-7"); 106.7 (C-6); 107.4 (C-10a); 107.6 (C-9a); 111.1 (C-8); 112.6 (C-2); 119.5 (C-4); 125.1 (C-2" and C-6"); 128.6 (C-2' and C-6'); 128.8 (C-3" and C-5"); 129.2 (C-3' and C-5'); 136.4 (C-1' and C-4"); 137.4 (C-7); 138.3 (C-4'); 140.8 (C-1"); 151.9 (C-3); 154.6 (C-4a); 155.5 (C-5a); 159.8 (C-1); 161.2 (C-9); 185.8 (C-10). ESI-MS *m/z* 421 [M−18+H]^+^, 443 [M−18+Na]^+^, 459 [M−18+K]^+^. HR-ESIMS (positive ion): *m/z* calcd. for C_28_H_20_O_5_ [M]^+^, 438,1467; found, 438.1477.

*6,8,13-Trihydroxy-2-methyl-13-(4-methyphenyl)indeno[1,2-c]xanthen-7(13H)-one* (**12b**). This compound was obtained by the treatment of a solution of (*E*)-8-hydroxy-4-(4-methylbenzylidene)-3-(4-methylphenyl)-3,4-dihydro-1*H-*xanthene-1,9(2*H*)-dione (**5b**, 57.6 mg, 0.14 mmol) in dried 1,24-trichlorobenzene (TCB) (10 mL) and in the presence of 100 mg molecular sieves with chloranil (2.0 equiv, 68.0 mg, 0.28 mmol). The mixture was then irradiated in an Ethos SYNTH microwave (Milestone Inc.) at 170 °C for 30 min. After this the TCB was removed by column chromatography using hexane as eluent and a semi-purified fraction was eluted with CH_2_Cl_2_. The desired compound **12b** was obtained by TLC purification using a mixture of hexane/CH_2_Cl_2_ (3:2) as eluent, as yellow crystals, 34% yield (20.7 mg). D.p. 145 °C; ^1^H-NMR (300 MHz, CDCl_3_, TMS) δ (ppm): 2.29 (s, 3H, 4"-CH_3_); 2.35 (s, 3H, 4'-CH_3_); 6.70 (dd, 1H, *J* = 0.8 and 8.3 Hz, H-6); 6.73 (dd, 1H, *J* = 0.8 and 8.3 Hz, H-8); 7.07 (s, 1H, H-2); 7.08 (d, 2H, *J* = 8.4 Hz, H-3",5"); 7.18 (d, 1H, *J* = 0.9 Hz, H-3'); 7.20–7.23 (m, 1H, H-5'); 7.33 (d, 2H, *J* = 8.4 Hz, H-2",6"); 7.50 (t, 1H, *J* = 8.3 Hz, H-7); 7.58 (d, 1H, *J* = 7.7 Hz, H-6'); 11.81 (s, 1H, 9-OH); 12.22 (s, 1H, 1-OH). ^13^C-NMR (75 MHz, CDCl_3_, TMS) δ (ppm): 21.1 (4"-CH_3_); 21.8 (4'-CH_3_); 83.1 (C-7"); 102.5 (C-2); 106.7 (C-10a); 107.2 (C-6); 107.6 (C-9a); 111.0 (C-8); 121.3 (C-6'); 125.1 (C-3', C-2" and C-6"); 128.9 (C-3" and C-5"); 130.1 (C-5'); 134.9 (C-1'); 137.0 (C-4"); 137.1 (C-7); 139.0 (C-4 and C-1"); 141.5 (C-4'); 150.2 (C-3 *); 151.5 (C-4a); 152.8 (C-2' *); 155.8 (C-5a); 161.2 (C-9); 163.7 (C-1); 185.3 (C-10) *May be interchanged. ESI-MS m/z 459 [M+Na]^+^. HR-ESIMS (positive ion): *m/z* calcd. for C_28_H_21_O_5_ [M+H]^+^, 437.1384; found, 437.1380.

### 3.3. Determination of the Reducing Power

The reductive potential of the compounds and standard was determined according to a previously described method [17]. An aliquot of the sample or standard (1.0 mL) at various concentrations (20–80 μg/mL in DMSO) was mixed with phosphate buffer (0.2 mol L^−^^1^, pH 6.6, 1.25 mL) and 1% potassium ferricyanide (1.25 mL). The mixture was incubated at 50 °C for 20 min. After cooling, 1.25 mL of 10% trichloroacetic acid was added and the mixture incubated at 50 °C for 10 min. 1.25 mL of this solution was mixed with distilled water (1.25 mL) and 0.1% iron(III) chloride (0.25 mL) and the absorbance measured at 700 nm using an appropriate blank. Assays were carried out in triplicate.

### 3.4. DPPH Radical Scavenging Assay

Antioxidant activity was assayed by the DPPH (1,1-diphenyl-2-picrylhydrazyl) radical scavenging assay using the Blois method modified [24]. Briefly, different volumes of the ethanolic solutions of each compound or standard (1–100 µM) were added fixed volume of DPPH ethanolic solution (400 µL of the solution 0.6 mM) and solvent (ethanol) to obtain, in each case, a fixed total volume. In each assay, a control was prepared, in which the compound or standard (quercetin and BHT) was substituted by the same amount of solvent. The mixture was shaken vigorously and left to stand for 30 min in the dark, and the absorbance was then measured at 517 nm. The capability to scavenge the DPPH radical was calculated using the following equation:

DPPH scavenging activity (%) = [(A_c_ − A_s_)/A_c_] × 100 (1)

where, A_c_ is the absorbance of the control and A_s_ is the absorbance of the compound or standard. All assays were carried out in triplicate and results expressed as EC_50_, *i.e.*, as the concentration yielding 50% scavenging of DPPH, calculated by interpolation from the % *vs.* concentration curve.

### 3.5. Microplate Assay for AChE Activity

The AChE activity was evaluated using the method described by Ellman *et al.* [25] and Ingkaninan *et al.* [26], modified. Briefly, 10 μL of 0.25 U/mL AChE from *Electrophorus electricus* (Sigma-Aldrich^®^ Chemical Company, St. Louis, MO, USA) was added to the wells containing sample in different concentrations, and allowed to incubate for 5 min. Reaction was started by the addition 5 μL of 3 mM 5,5'-dithiobis[2-nitrobenzoic acid] (DTNB,) and 5 μL of 75 mM acetylthiocholine iodide (ATCI; Fluka Chemicals, Buchs, Switzerland). The absorbance was then read at 415 nm every 2.5 min for 7.5 min in a Bio Rad Model 680 Microplate Reader (Bio-Rad Laboratories, Inc., Hercules, CA, USA). Percentage of enzymatic inhibition was calculated relatively to the control without inhibitor. Every experiment was done in quadruplicate. To determine IC_50_ values, EPA PROBIT ANALYSIS PROGRAM Version 1.4 was used.

## 4. Conclusions

In conclusion, (*E*)-3-aryl-4-benzylidene-8-hydroxy-3,4-dihydro-1*H-*xanthene-1,9(2*H*)-diones **5** can be successfully obtained by simple and efficient methodologies from (*E*,*E*)-3-cinnamoyl-5-hydroxy-2-styrylchromones **4**. These compounds seem to be good synthons towards new interesting xanthone derivatives and, with the appropriate substitution patterns, powerful antioxidants and AChEI. The combination of these activities and the inhibition type displayed by these compounds suggest that they may be the excellent templates for drugs to be used in the prevention and treatment of neurodegenerative diseases.
